# Isolated rupture of the corpus spongiosum with urethral injury diagnosed by pre‐surgical MRI

**DOI:** 10.1002/iju5.12559

**Published:** 2022-11-22

**Authors:** Shuhei Yokoyama, Ichiro Tsuboi, Kohei Ogawa, Saori Yoshioka, Yusuke Kobayasi, Hirochika Nakajima, Taichi Nagami, Seigen Yamasaki, Koichiro Wada

**Affiliations:** ^1^ Department of Urology, Faculty of Medicine Shimane University Japan; ^2^ Department of Urology Kyushu Rosai Hospital Japan

**Keywords:** corpus spongiosum, direct incision, MRI, penile fracture

## Abstract

**Introduction:**

Penile fracture is typically defined as the rupture of the corpus cavernosum.

**Case presentation:**

A 61‐year‐old man presented with swelling, pain, and bruising of his penis, along with gross hematuria. He reported that he sustained this injury while having sex with his wife. We suspected a penile fracture and obtained magnetic resonance imaging, which showed a rupture of the ventral corpus spongiosum and clarified the appropriate approach for repair. We used a direct transverse incision to repair both the urethral injury and the corpus spongiosum. Surgery went well, without any significant intraoperative or postoperative complications. We removed the urinary catheter on postoperative day 8, and cystoscopy showed no urethral stenosis on postoperative day 17. The patient's postoperative erectile function was the same as before his injury.

**Conclusion:**

Magnetic resonance imaging was useful for detect the site of rupture. Ventral direct transverce incision made him a good clinical course.

**Abbreviations & Acronyms**
CC: corpus cavernosum
CS: corpus spongiosum
MRI: magnetic resonance imaging
PF: penile fracture
US: ultrasonography

**Keynote message**

Penile fracture is a rare urological emergency disease. Usually, the site of rupture is corpus cavernosum although we experienced the isolated ruputer of the corpus spongiosum with urethral injury. Magnetic resonance imaging was useful for detecte the rupture. The imaging also enabled us to perform the direct transverse incision.

## Introduction

Penile fracture (PF) is a rare urologic emergency. It is defined as a tear of the tunica albuginea of the corpus cavernosum (CC) caused by external force applied to the erect penis, resulting in deformation, bending, and swelling of the penis.^1^ In rare cases, patients may rupture the corpus spongiosum (CS) alone, without rupturing the CC. To our knowledge, there are few reports of an isolated ruptured CS with urethral injury. Herein, we report our experience with a patient who had a rupture of the CS with urethral injury that was easily detected on magnetic resonance imaging (MRI). Preoperative imaging was useful in choosing the direct transverse incision approach to repair.

## Case presentation

**Fig. 1:**
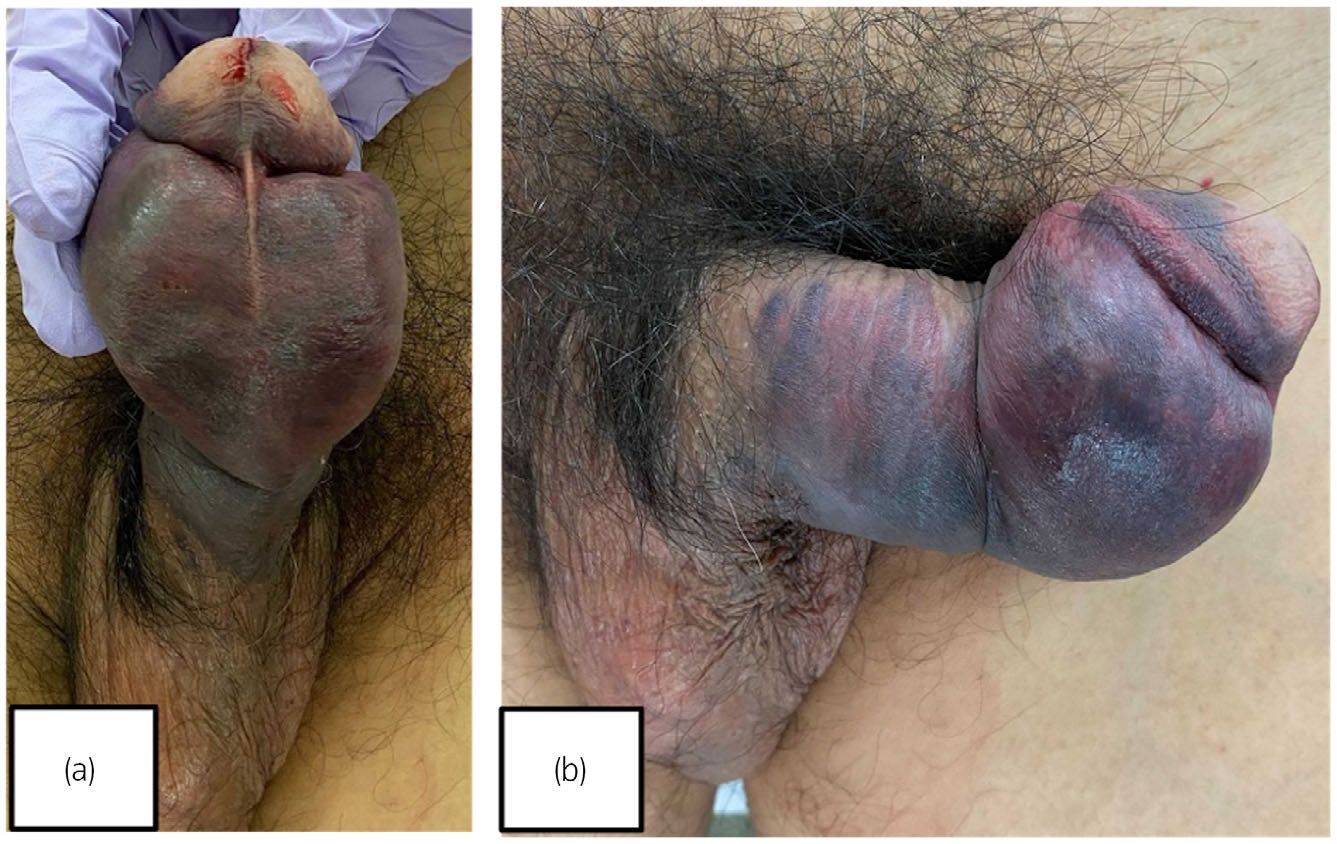
(a) and (b) showed that the penis is grossly deformed with an apparent subcutaneous hematoma.

A 61‐year‐old man was injured when his penis slipped out of his wife's vagina during penovaginal intercourse with penetration from behind (“doggy style”). His physician treated the penile swelling with hemostatic agents, but his symptoms worsened. When he presented to our institution, 13 hours had passed. Physical examination showed a swollen penis with an “eggplant” deformity caused by a hematoma on the distal ventral surface of the penis; he also had gross hematuria visible at the external urethral orifice (Fig. 1). His past medical history was significant for benign prostatic hyperplasia, for which he was taking tadalafil.

**Fig. 2:**
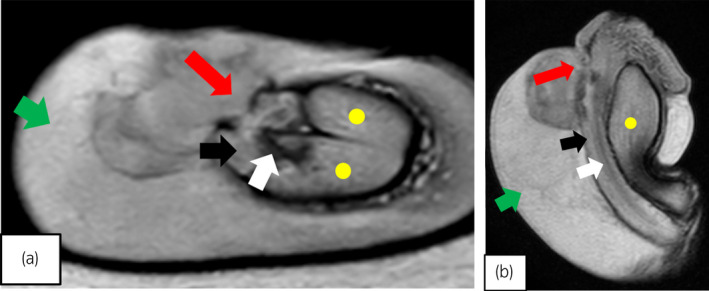
T2‐weighted magnetic resonance imaging shows an injury of the ventral tunica albuginea of the corpus spongiosum. (a) axial image. (b) sagital image. Green arrows: hematoma; red arrows: rupture of the corpus spongiosum; black arrows: corpus spongiosum; Yellow dots: corpus cavernosum; white arrows: urethra.

**Fig. 3:**
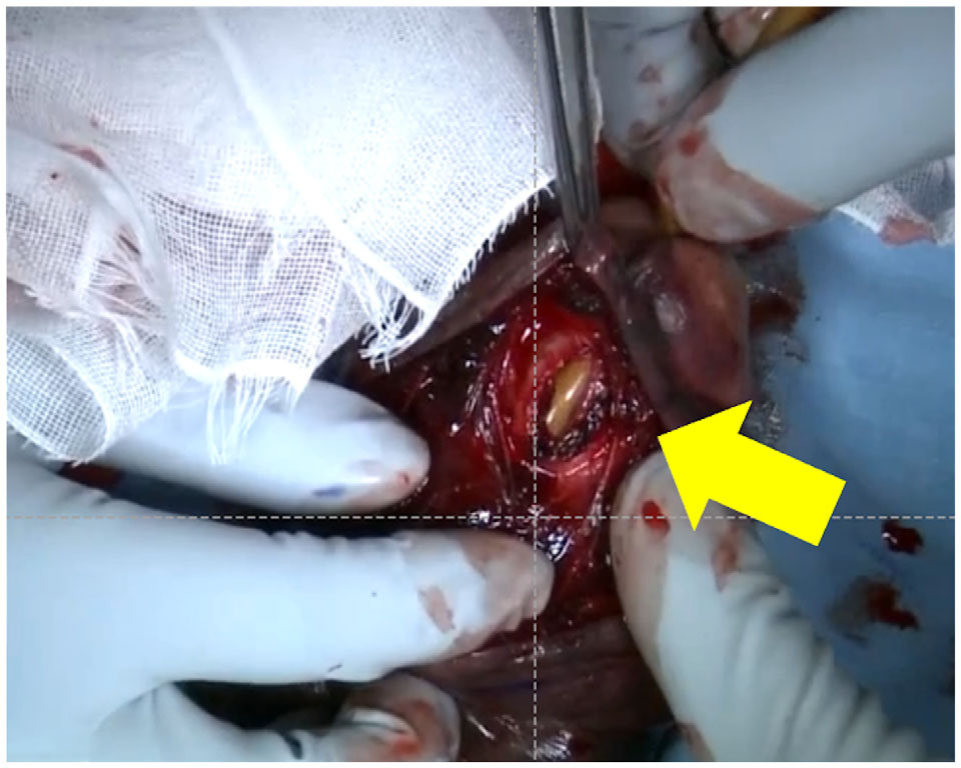
The urinary catheter is visible through the damaged urethra.

We ordered an immediate MRI; T2‐weighted imaging revealed an isolated tear of the ventral tunica albuginea of the CS (Fig. 2). We diagnosed a rupture of the CS with urethral injury and proceeded to the operating room. A 5‐cm transverse incision of the epidermis just above the subcutaneous hematoma allowed us to remove as much blood and clot as possible. A 2‐cm longitudinal injury of the tunica albuginea of the CS was visible, and the anterior urethra was ruptured; a 14‐Fr urethral catheter was visible through the defect in the urethra (Fig. 3). We repaired the tunica albuginea of the CS and the urethral mucosa using interrupted 3–0 and 4–0 absorbable sutures, respectively.

**Fig. 4:**
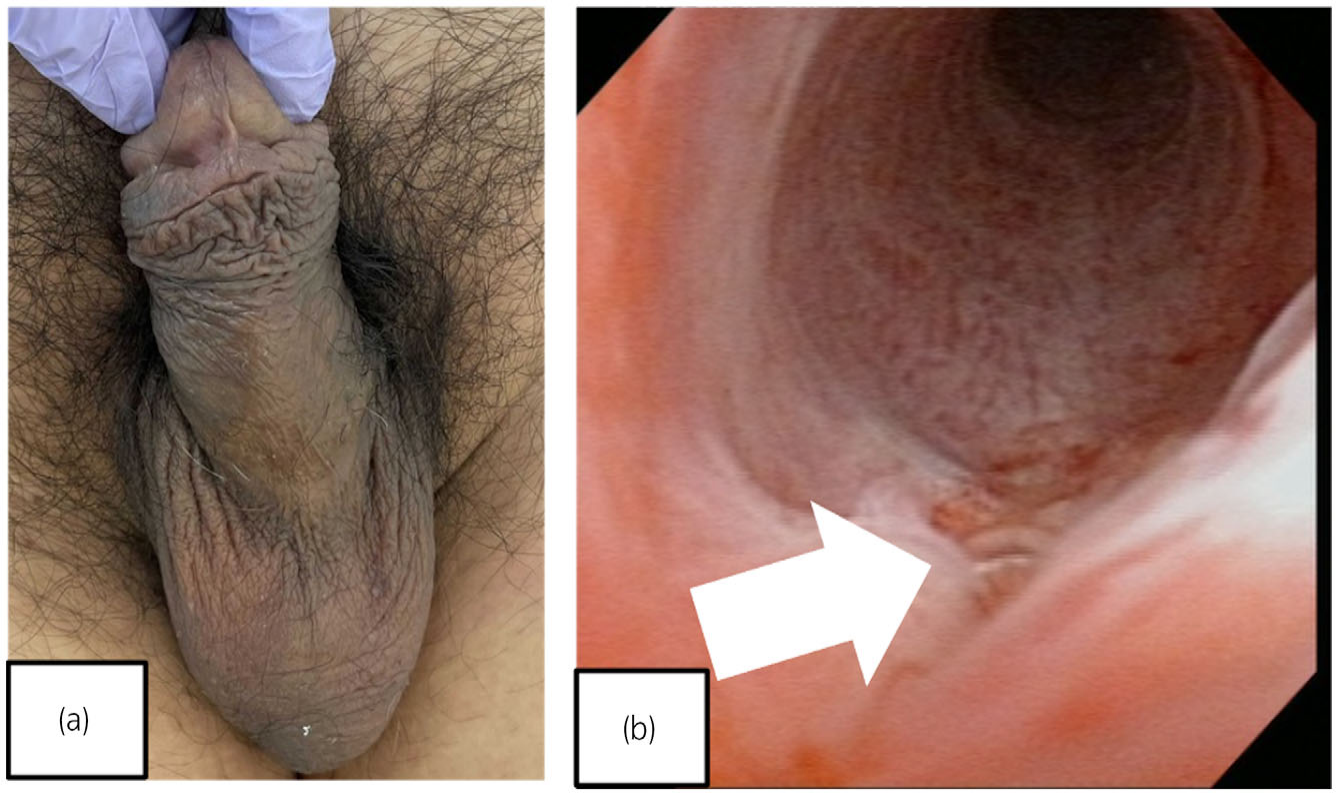
(a) Postoperative day 17: the penile hematoma and deformity are improved. (b) Cystoscopy 6 weeks after surgery reveals only mild edema. White arrow: the 4‐0 polydioxanone suture.

The patient's postoperative course was unremarkable. We removed the urinary catheter on postoperative day 8, and the patient was discharged home. On postoperative day 17, he returned for follow‐up. There was no discoloration or swelling of the penile shaft, and his erectile function had returned to his pre‐injury status. Cystoscopy showed no obvious urethral stenosis or injury except for residual edema (Fig. 4).

## Discussion

A rare urologic emergency, PF usually occurs during sexual intercourse. The site of fracture is the tunica albuginea of a unilateral CC; urethral injury occurs in about 20% of patients. An isolated tear of the CS with urethral injury is more rare. Common symptoms are the same as those of a PF: a fracture sound, pain, swelling, and bruising. When gross hematuria is observed, urethral injury should be suspected.^1^, ^2^ Our patient had a ruptured CS with a urethral tear but without injury of the CC. The mechanism of this isolated injury is unknown.^3^

The diagnosis of PF can usually be made by history and physical examination; patients report a cracking sound, and there is pain, swelling, and visible hematoma of the penis. Rapid surgical management is required for suspected PF, especially when urethral injury is likely. Therefore, the role of MRI and ultrasonography (US) is controversial. El‐Assmy et al reported that penile US is useful for ruling out PF or identifying the location of the fracture. The AUA guidelines recommend consideration of MRI when US is inconclusive for PF.^4^ Spiesecke et al reported the sensitivity and specificity of US (71.4% and 100%) and MRI (91.9% and 97.2%).^5^ However, US is highly dependent on the skill of the operator, while MRI is not relatively dependent on it. In Japan, MRI can be performed and is relatively easy and quick. Thus, we decided to obtain a MRI before moving to surgical management: this allowed us to easily verify the site of the rupture.

Surgical repair is the treatment of choice for PF. Conservative management results in sequelae such as erectile dysfunction, deformity, and curvature of the penis. The repair procedure removes the hematoma and reapproximates the damaged tunica albuginea. However, the optimal site of skin incision and method of approach are controversial. The incision site is determined by the situation. Commonly used approaches include the circumferential incision; the direct incision, which is directly over the tunica albuginea tear site; and the penoscrotal incision, which runs longitudinally over the midline of the penile root.^1^ Because we had a preoperative MRI to consult, we were able to choose the direct transverse incision and easily visualize the injury.

## Conclusion

We experienced a patient who had a ruptured CS with urethral injury. Preoperative MRI helped us detect the precise site of the rupture and enabled us to choose the correct incision to repair this patient's injury.

## Author contributions

Shuhei Yokoyama: Conceptualization; writing – original draft. Ichiro Tsuboi: Conceptualization; writing – review and editing. Kohei Ogawa: Writing – review and editing. Saori Yoshioka: Writing – review and editing. Yusuke Kobayashi: Writing – review and editing. Hirochika Nakajima: Writing – review and editing. Taichi Nagami: Writing – review and editing. Seigen Yamasaki: Writing – review and editing. Koichiro Wada: Supervision; writing – review and editing.

## Conflict of interest

The authors declare no conflict of interest.

## Approval of the research protocol by an Institutional Reviewer Board

Not applicable.

## Informed consent

Informed consent was obtained from the patient.

## Registry and the Registration No. of the study/trial

Not applicable.
